# Research on Bio-Inspired Decussated Bamboo-Fiber-Reinforced Epoxy Composites: The Effect of Vertical Fiber Proportion on Tribological Performances

**DOI:** 10.3390/polym17202765

**Published:** 2025-10-15

**Authors:** Heng Xiao, Hao Yi, Zijie Zhou, Ningfeng Wu, Shengwei Liang, Lei Ma, Wen Zhong

**Affiliations:** 1 School of Mechanical Engineering, Xihua University, Chengdu 610039, China; heng@mail.xhu.edu.cn (H.X.); 18681737550@163.com (H.Y.); 2 Research Institute of Xihua University (Yibin), Yibin 644000, China

**Keywords:** bamboo fibers, bio-inspired decussated structure, tribological properties, epoxy composites, friction materials

## Abstract

Bamboo fiber is a prime green fiber due to its renewability, biodegradability, and high specific strength. Bamboo-fiber-reinforced epoxy (BFRE) composites have seen extensive use and shown great promise for natural biofiber-reinforced friction materials. Inspired by the decussated fiber alignment of bovine enamel, this study investigated how fiber orientation influences the tribological properties of BFRE composites. Specifically, the proportion of fibers oriented vertically to the surface was varied at seven levels: 0%, 25%, 33%, 50%, 67%, 75%, and 100%. The tribological performance was assessed through wear reciprocating testing and microscopic morphological characterization techniques. Results indicate that the bio-inspired fiber decussation can reduce the wear loss of the BFRE composites. Among all bio-inspired BFRE composites, BFRE composites with 67% vertical fibers achieve the best wear resistance. The vertical fibers in the BFRE composites can withstand pressure to provide a “compression–rebound” effect, while the parallel fibers can resist shear stress. The decussated structure inhibits crack initiation and propagation during wear and promotes transfer film formation, reducing wear loss. The findings expand understanding of the correlation between the bovine-tooth-like decussated structure and its tribological mechanisms, thereby offering essential guidance for the biomimetic design of high-performance BFRE composites for friction material application.

## 1. Introduction

General friction materials pose a significant environmental and health challenge: during both the manufacturing and operational processes, they generate sub-10 μm particles that disseminate into the environment, potentially threatening human and animal health [1]. With the accelerating global transition toward low-carbon and green development, the development of environmentally friendly and high-performance friction materials has emerged as a critical research priority. Renowned for their high specific strength and modulus, low density, facile biodegradability, and renewable nature, bamboo fibers have gained considerable attention as a prime example of green fibers [2,3,4]. Composites reinforced with bamboo fibers are already utilized in transportation, construction, and sports sectors, demonstrating their potential as sustainable alternatives for natural biofiber-reinforced friction materials.

In early studies on bamboo-fiber-reinforced composites, Mandal et al. [5] partially replaced glass fibers with bamboo fibers to prepare glass/bamboo-fiber-reinforced composites and found that substituting 25% of the glass fibers retained the original mechanical properties. Furthermore, incorporating appropriate amounts of wool and jute fibers into bamboo fiber composites was found to improve their thermal stability [6]. Moreover, surface modification studies revealed that increasing bamboo fiber surface roughness improves the frictional performance of composites under tensile loads and high-temperature conditions [7]. Studies on material composition revealed that bamboo fiber composites with brittle polymer matrices exhibit excellent toughness [8]; among them, epoxy-resin-based composites exhibit significantly higher tensile and flexural strength than their polyester-based counterparts [9]. Epoxy resin, serving as the matrix in composite materials, possesses outstanding mechanical properties. Its composition includes a significant quantity of reactive oxygen-containing groups and hydroxyl functional groups, enabling it to form robust bonds with fibers, which significantly enhances the overall mechanical strength of the composite material [10]. Incorporating bamboo fibers into epoxy resin composites significantly further enhances their strength and stiffness [11]. But the tribological performance of the bamboo-fiber-reinforced epoxy (BFRE) composites remains inadequate in the friction material field [12].

In recent years, the fiber aspect ratio and alignment characteristics have been found to significantly influence the tribological properties of composites, which provided numerous inspirations for developing novel BFRE composites to apply in the friction material field. As the fiber aspect ratio increases and/or the degree of alignment improves, the bamboo fibers become more difficult to pull out under certain loads, and thus the mechanical properties of the composites, such as tensile strength and Young’s modulus, are enhanced [13]. In terms of fiber orientation research, Nirmal et al. [14] and Oliver et al. [15] investigated the effects of fiber orientation and cross-angle on the frictional behavior of the composites. They found that improving fiber alignment enhances surface wear resistance; however, when the fiber cross-angle reaches 45°, surface shear stress increases and wear resistance decreases. Bamboo, as a natural composite material with a thin-walled parenchyma matrix and reinforcing bamboo fibers, exhibits a clear positive correlation between mechanical properties and fiber distribution density. Its strength increases progressively from the inner to the outer layer with the change in fiber density and orderliness [16]. Additionally, the ordered and layered arrangement of bamboo fibers enables interlayer slippage under load, which effectively prevents crack propagation along fracture surfaces [17]. But when fiber alignment in the node region is lower than that in the internode region, tensile strength decreases significantly [18]. These findings suggest that, in addition to surface modification and compositional adjustment, fiber orientation is a critical factor in enhancing the frictional performance of BFRE composites. However, current studies on fiber orientation remain limited, with relatively simplistic design strategies and insufficiently comprehensive evaluations of frictional performance.

In engineering applications, friction materials are required to simultaneously possess high mechanical strength and favorable tribological properties—namely, high friction coefficients and low wear rates—two characteristics that are often inherently contradictory. Interestingly, certain natural biological composites, such as animal teeth, exhibit remarkable wear resistance, mechanical strength, and surface friction behavior as a result of their evolutionary adaptation to chewing. Notably, a decussating structure of fiber alignment was discovered in the enamel of bovine teeth, which not only resists long-term wear but also suppresses crack propagation, thereby maintaining structural integrity [19,20]. The nanofibers horizontally orientated on the tooth surface provide enhanced corrosion resistance and anti-crack properties [20], while nanofibers orientated consistent with the loading direction enhance the load-bearing capacity of the surface, thereby effectively resisting wear [21]. Therefore, this study focuses on the enhancing effect of the decussating structures inspired by bovine teeth on the tribological properties of BFRE composites. Specifically, the effects of the proportion of the fibers perpendicular to the testing surface on its surface tribological behaviors and wear resistance are the major concern. This study aims to reveal the influence of bamboo fiber alignment, especially the fiber orientation, on the tribological behaviors of the bio-inspired BFRE composites and then contribute to providing theoretical guidance for the development of high-performance and green friction materials.

## 2. Materials and Methods

### 2.1. Material and Preparation Process

**Figure 1:**
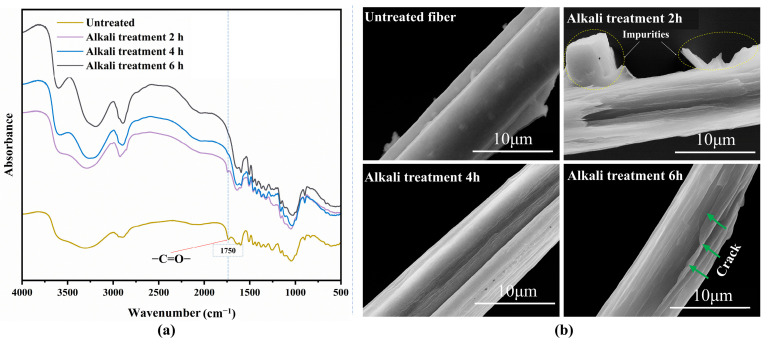
Characterization of bamboo fibers with different alkali treatments: (**a**) FT-IR spectra of bamboo fibers following alkali treatments; (**b**) SEM images of bamboo fiber topographies.

The bamboo fibers used in this study were derived from moso bamboo (*Phyllostachys edulis*) (Huazhao Technology Co., Ltd., Chengdu, China). The bamboo was 2–3 years old, with the outer green layer removed. The average length of the bamboo fibers used was 8 ± 2.3 mm, with an average equivalent diameter of 80 ± 12 µm. To enhance interfacial bonding with the matrix, the fibers underwent an alkaline treatment. Sodium hydroxide (NaOH) (Aladdin Co., Ltd., Shanghai, China), owing to its hydration layer, can readily penetrate the internal pores of bamboo fibers, dissolving hemicellulose and lignin [22,23]. Following alkaline treatment, the condition of the fibers was analyzed using a scanning electron microscope (SEM) (KYKY-SEM6900, Zhongke Keyi Co., Ltd., Beijing, China) and a Fourier Transform Infrared Spectrometer (FT-IR) (L1600400 Spectrum TWO, PerkinElmer, UK). As shown in Figure 1a, FT-IR results reveal a distinct peak in the untreated sample and a light peak in the 2 h treated sample at approximately 1730 cm^−1^, which corresponds to hemicellulose and lignin [24]. It indicates that the hemicellulose and lignin of the bamboo fiber were removed after 4 h of alkali treatment. In Figure 1b, significant crack damage can be observed in the SEM image of the fibers after 6 h alkali treatment. Based on the preliminary experiment results and the literature [25], treatment using 4% NaOH solution at 60 °C for 4 h, followed by drying at 40 °C for 24 h, is the most appropriate for this study and thus was selected as the optimal condition for preparing the bio-inspired BFRE composites.

**Figure 2:**
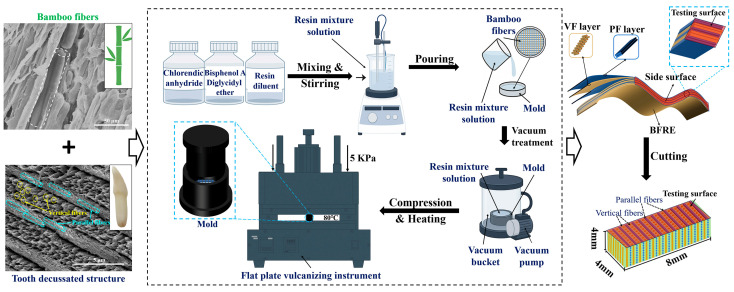
Schematic diagram of the source of inspiration and the sample preparation process.

Inspired by the decussated fiber alignment of bovine teeth, specimens with different proportions of fiber alignment were prepared according to the procedure shown in Figure 2. The resin matrix and curing agent used in this study are bisphenol-A-diglycidyl-ether (density: 1.167–1.171 g/mL; purity ≥ 85%) and chlorosilane anhydride (density: 1.73 g/cm^3^; purity ≥ 95%), respectively, both supplied by Aladdin Co., Ltd., Shanghai, China. An epoxy-active diluent, HK-66 alkylene glycidyl ether, provided by Yuanbang Chemical Materials Co., Ltd. (Zhangjiagang, China), was used as both a diluent and catalyst. In priority, all the mass fractions of each component (see Table 1) in the BFRE composites, including bamboo fibers, were ensured using an analytical balance (RC-FA-2004EN, Ruicheng Yongchuang Technology Co., Ltd., Beijing, China). They were mixed and stirred for 10 min using an electric stirrer (LC-ES-200SH, Lichengbangxi Instrument Technology Co., Ltd., Shanghai, China), followed by vacuum degassing to remove entrapped air bubbles, to obtain a resin mixture solution.

The mold used in the fabrication, with dimensions of Φ40 × 10 mm, was made of aluminum alloy. Before casting, the inner surface of the mold was first lined with Teflon high-temperature-resistant tape (Wangxing Tape Co., Ltd., Shenzhen, China) and then coated with a high-temperature oil-based epoxy release agent. Following this, the bamboo fibers, after being weighed, were arranged in the mold by hand to ensure the fiber orientation layer by layer. And the thickness of the layers was controlled to obtain different proportions of the fiber orientations in the BFRE composites. Afterwards, the resin mixture solution was slowly poured into the mold, followed by 30 min of vacuum degassing treatment. A flat vulcanizing press (Model ST-15YP, Lu Gong Precision Instrument Co., Ltd., Kunshan, China) was used to further remove the residual air bubbles in the mold with 5 kPa of pressure and 80 °C of temperature for 8 h to fully cure the composites. After demolding, the specimens were cut into 8 × 4 × 4 mm blocks, with the side surface serving as the testing surface. Based on the orientation of bamboo fibers, the fibers perpendicular to the testing surface are termed “vertical fibers (VFs)”, while those parallel to the surface are termed “parallel fibers (PFs)”. Specimen groups were distinguished by vertical fiber proportion (VFP): VFP0% (0% vertical), VFP25% (25% vertical), VFP33% (33% vertical), VFP50% (50% vertical), VFP67% (67% vertical), VFP75% (75% vertical), and VFP100% (100% vertical). The specimen blocks were subsequently encapsulated within a 20 × 10 × 8 mm base resin (self-curing resin). To obtain flat surfaces, all samples were ground with silicon carbide paper of 500, 1000, and 2000 grade and then polished with 0.5 µm diamond suspension until the surface roughness was less than 0.01 mm, which was measured using a laser confocal microscope (LCSM) (VK-X1000, KEYENCE, Osaka, Japan). The surface morphologies of each group sample were examined using an ultra-depth-of-field 3D microscopy system (RS-V1, Futuo Optoelectronic Precision Instruments Co., Ltd., Wuhan, China).

### 2.2. Hardness Tests

**Figure 3:**
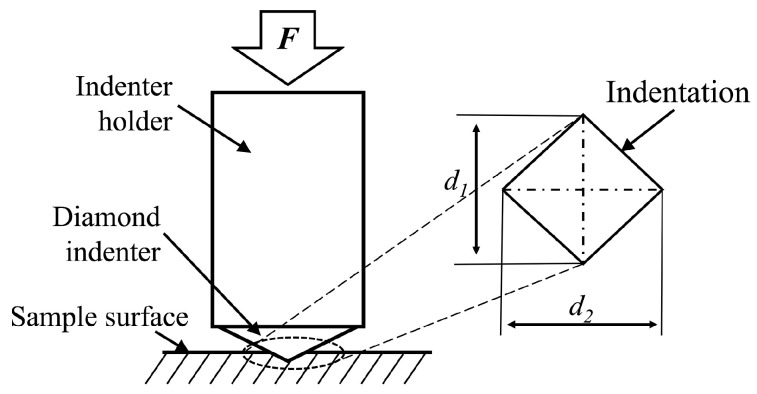
Schematic diagram of the Vickers hardness test.

The hardness of the bio-inspired BFRE composite surfaces was measured by a Vickers hardness tester (HV-1000, Hengxinjie Technology Co., Ltd., Shenzhen, China) using a diamond indenter with a radius of 200 µm under a load of 0.49 N, with a loading duration of 20 s, as shown in Table 2. Ten samples for each group of the bionic BFRE composites were used, and 12 indents were conducted for each sample. Based on the morphologies of the indentations observed by the aforementioned 3D microscopy system and SEM, the measurements of Vickers hardness (HV) were calculated using Equation (1):

(1) $$HV=0.1891\times \frac{F}{d^{2}}$$

where *F* (N) is the initial testing force and *d* (mm) is the arithmetic mean of *d*_1_ and *d*_2_, which are obtained through the indentation size (Figure 3).

### 2.3. Tribological Tests

**Figure 4:**
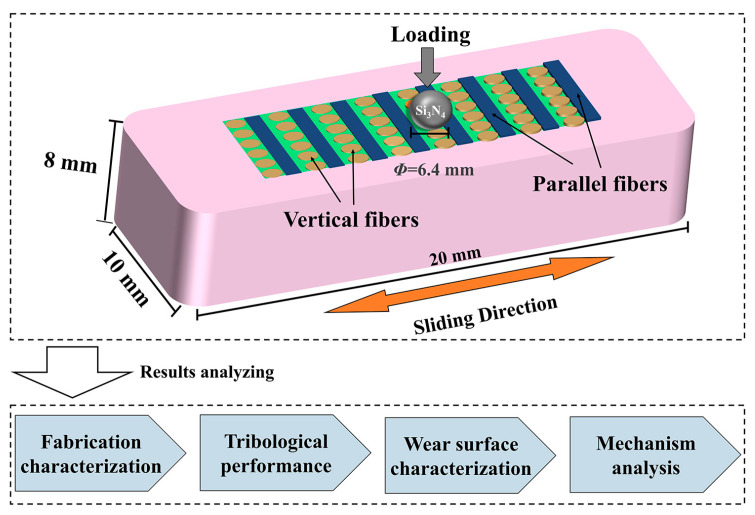
Schematic diagram of the wear testing.

A multifunctional friction testing machine (MWF-02, Sichuan Jiaoyang Technology Co., Ltd., Chengdu, China) with a reciprocating module was used to investigate the effect of VF proportion on the tribological behaviors of the BFRE composites under dry sliding conditions. A Si_3_N_4_ ceramic ball, due to its excellent mechanical properties [26], was used as the counterface material. The Si_3_N_4_ ceramic ball was controlled to slide transversely across the parallel fibers during the tests, as shown in Figure 4. A reciprocating stroke of 4 mm and a frequency of 5 Hz (0.021 m/s) were applied in the tests, with normal loads of 5 N, 10 N, and 20 N applied. Each test was conducted for 30 min, with data recorded at 0.01 s intervals. Twelve samples were used for each group under different loading conditions (Table 2).

After the tribological tests, each sample was examined using the LCSM, while the microscopic features of the wear marks were observed with the SEM. The specific wear rate (W) was then calculated based on the measured wear volume using Equation (2):

(2) $$W=\frac{V}{LS}$$

where *V* (mm^3^) is the wear volume, *L* (N) is the normal load, and *S* (m) is the total sliding distance. The wear volume was obtained directly through the data processing software within the LCSM, while the total sliding distance was calculated as the product of stroke, time, and frequency.

For each sample type, to identify significant differences in either hardness or wear rate of the sample surface under different conditions, statistical analysis was performed using repeated measures analysis of variance (ANOVA). Differences meeting the level of significance (*p* < 0.05) were considered significant.

## 3. Results

### 3.1. Fabrication Characterization

**Figure 5:**
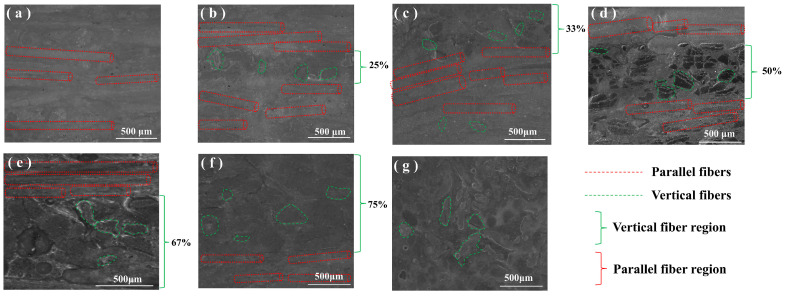
Surface morphologies of different BFRE composites: (**a**) VFP0%; (**b**) VFP25%; (**c**) VFP33%; (**d**) VFP50%; (**e**) VFP67%; (**f**) VFP75%; (**g**) VFP100%.

To ensure successful fiber arrangement fabrication, the surface morphologies of all groups were observed and compared, as shown in Figure 5. On the surfaces of the BFRE composites, fiber bonding was observed significantly due to fiber compression during sample preparation. As can be seen, all bio-inspired BFRE composites consist of VFs and PFs arranged in an orderly alternating pattern (Figure 5b–f). Fibers at the interface of the VF and PF regions are decussated with an angle of 90°. The VF proportion of each sample surface was finely controlled. Some surface defects can be observed, and the number of defects on the sample reduces with the increase in VF proportions.

### 3.2. Vickers Hardness

**Figure 6:**
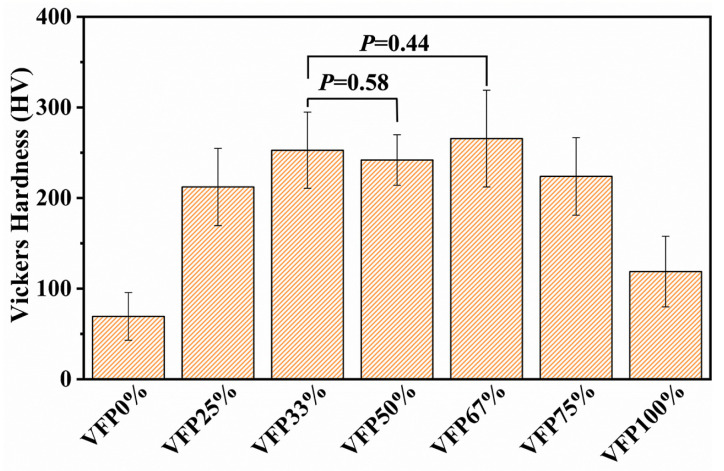
Surface hardness of different BFRE composites (*p* < 0.05).

Figure 6 shows the surface Vickers hardness of the BFRE composites. The average hardness of each group follows the order VFP67% > VFP33% > VFP50% > VFP75% > VFP25% > VFP100% > VFP0%, but there are no significant differences between the VFP67%, VFP33%, and VFP50% groups (*p* < 0.05).

### 3.3. Tribological Performances

**Figure 7:**
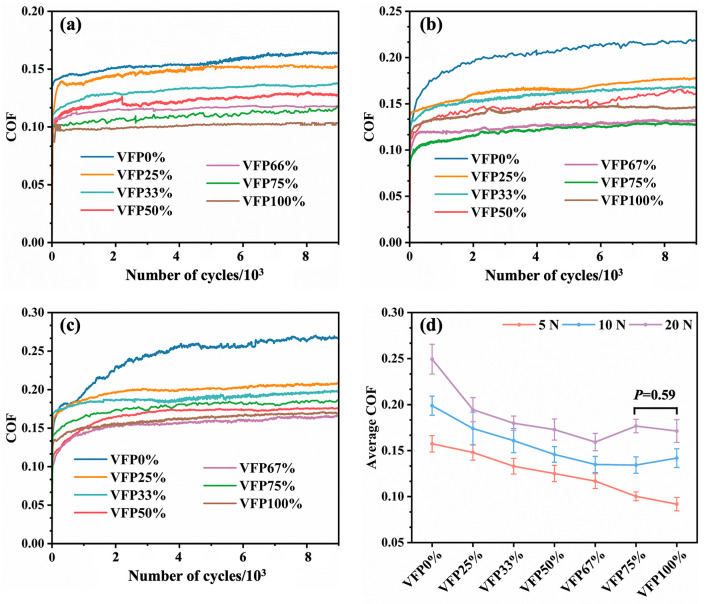
The COF curves and average statistic results of the BFRE composites under different loading conditions: (**a**) 5 N; (**b**) 10 N; (**c**) 20 N; (**d**) average COF (*p* < 0.05).

Figure 7 illustrates the coefficient of friction (COF) curves and average statistical results of the BFRE composites under varying loads. As depicted, the friction behavior can be divided into two distinct phases: the “break-in phase” and the “steady-state phase”. As shown in Figure 7a–c, nearly all types of BFRE composites exhibit an initial sustained increase followed by stabilization across different load conditions. And the time required for the friction process to reach a steady-state progressively increases with the load, while the amplitude of fluctuations in the friction coefficient curve gradually diminishes. Note that the COF value of the steady state rises with the load increasing for the same group. Based on the stable stage of each COF curve, the average COFs of different BFRE composites were calculated and are shown in Figure 7d. Under all loading conditions, the average COF gradually decreases as the VF proportion increases on the surfaces of the BFRE composites. However, under the load of 10 N and 20 N, the average COF reaches the lowest level when the VF proportion is 67%, but afterward, it increases with the further increase in the proportion. It is worth noting that under a 20 N load condition, there is no significant difference between VFP75% and VFP100% (*p* < 0.05).

**Figure 8:**
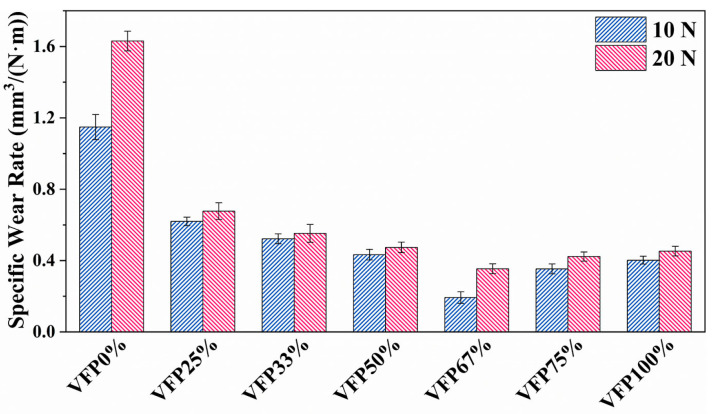
The specific wear rates of different BFRE composites (*p* < 0.05).

Due to the excessively low level of wear under 5 N load, the measurements of the wear volume are nearly impossible, and thus only the specific wear rates under the loads of 10 N and 20 N are calculated and shown in Figure 8. As can be seen, the specific wear rates of BFRE composites under the loads of 10 N and 20 N sharply decrease from 1.63 ± 0.05 and 1.14 ± 0.07 to 0.62 ± 0.02 and 0.67 ± 0.04, respectively, with VFR increasing from 0% to 25%. And they reach the lowest values (0.19 ± 0.03 and 0.35 ± 0.02, respectively) when VFR is 67%.

**Figure 9:**
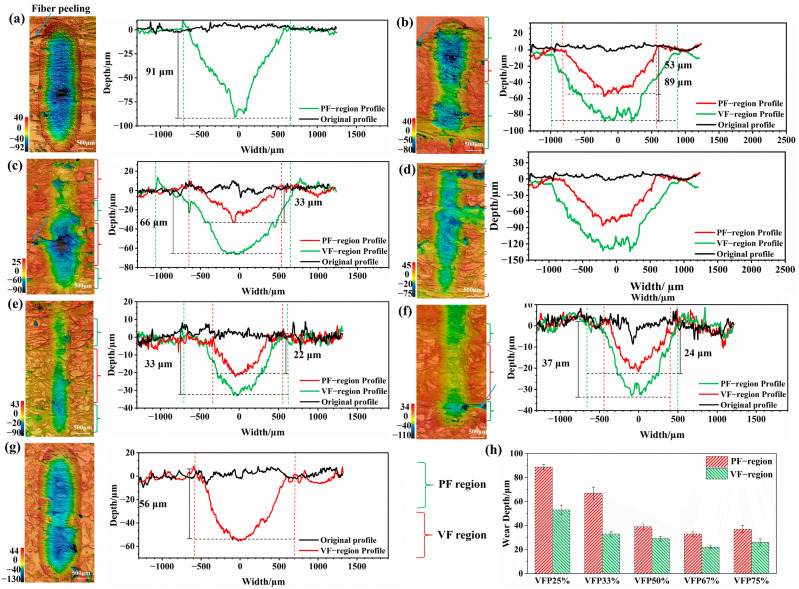
Three-dimensional surface morphologies and surface profiles of the worn surfaces under the load of 20 N for different BFRE composites: (**a**) VFP0%; (**b**) VFP25%; (**c**) VFP33%; (**d**) VFP50%; (**e**) VFP66%; (**f**) VFP75%; (**g**) VFP100%. (**h**) The wear depths in PF and VF regions (*p* < 0.05).

Considering that the phenomena under the load of 20 N are more pronounced compared to those under the load of 10 N, only the 3D surface morphologies and surface profiles in different regions of the worn surfaces of different BFRE composites under the load of 20 N are analyzed and shown in Figure 9. As shown in Figure 9a, VFP0% has the maximum wear depth, reaching 91 μm, among all groups. The worn surface exhibits visible traces of fiber peeling. As the proportion of VFs gradually increased from 0% to 67%, the maximum wear depth gradually decreased (Figure 9b–e). And it should be noted that the maximum wear depth in the PF regions is significantly higher than that in the VF regions for all bio-inspired BFRE composites. For VFP25% (Figure 9b), the PFs exhibit significant fiber peeling and damage. On the worn surfaces of the BFRE composites where the VF proportion exceeds 50%, it becomes increasingly difficult to observe fiber peeling (Figure 9e,f). For VFP100%, all fibers are vertical, achieving a maximum wear depth of 64 μm with a smooth worn surface (Figure 9g). In Figure 9h, the wear depths in PF regions are always larger than those in VF regions for each group. Simultaneously, the wear depths in the PF and VF regions are increasingly smaller with the VF proportion increasing from 25% to 67%, and they reached the lowest value (33 ± 2.13 and 22 ± 1.33, respectively) for VFP67%. With the VF proportion increasing to 75%, the wear depths increased in both the PF and VF regions.

### 3.4. Micro-Topographies of the Worn Surfaces

**Figure 10:**
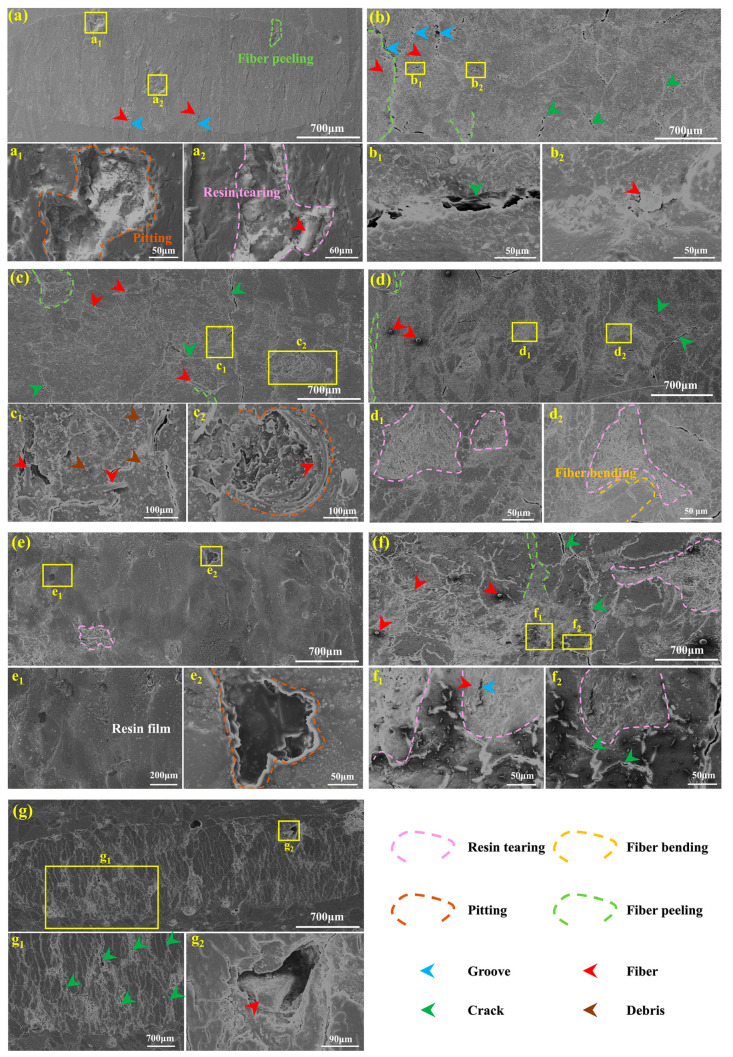
Typical microscopic morphologies of the worn surfaces of different BFRE composites under the load of 20 N: (**a**) VFP0%; (**b**) VFP25%; (**c**) VFP33%; (**d**) VFP50%; (**e**) VFP67%; (**f**) VFP75%; (**g**) VFP100%; a1–g1 and a2–g2 represent the enlarged images of (**a**–**g**), respectively.

To better understand the tribological behaviors of different BFRE composites, SEM morphologies of the worn surfaces were taken and are shown in Figure 10. The worn surfaces of VFP0% exhibit grooves formed by extensive resin tearing on both sides of the PFs, accompanied by fiber delamination (Figure 10a). With the VF proportion gradually increasing from 25% to 67%, the formation and propagation of cracks on the worn surface progressively multiplied. For VFP25%, as shown in Figure 10b, numerous individual fibers or random mixtures of fibers and resin wear debris can be easily observed on the worn surface, as well as in the wear pits (Figure 10b). As the VF proportion increased to 33%, fiber nodes of VFs began to appear on the worn surface (Figure 10c). The primary defect of the worn surface of VFP50% is resin tearing (Figure 10d), and some bent fibers can be observed near the areas of resin tearing. But there are no grooves near the bent fibers. As the VF proportion increased to 67% (Figure 10e), a resin film layer, with only minor pitting visible, appeared on the worn surface, instead of fiber peeling. With the VF proportion further increasing, the resin film layer disappeared, with a number of defects appearing on the worn surface accordingly. For VFP75% (Figure 10f), severe damage can be easily observed on the worn surface, including resin tearing, fiber peeling, and cracks. When the VF proportion increases to 100%, all fibers on the surface are vertical (Figure 10g), and cracks become the main damage behavior.

## 4. Discussion

Generally, the functional superiority of tooth enamel is tightly associated with its unique microstructure, especially highly aligned hydroxyapatite (HAP) fibers and enamel rods and their synergic effect [20]. On this basis, many bio-inspired materials have been prepared by controlling fiber orientations and have been reported to have the potential to be used as artificial dental materials and even in other applicable fields [21]. Thus, bovine tooth enamel, known for its excellent anti-wear and crack-resistance properties [20], was selected as the bionic model in this study. As shown in Figure 3, on the occlusal surface of bovine enamel, elongated semi-keyhole-like rods align in rows between the sheets of inter-rod enamel, and the HAP fibers in the rods are perpendicular to the occlusal surface, while those in the inter-rod enamel are parallel to it. That is, the fibers in the two zones are in decussation. Inspired by this, a novel BFRE composite has been fabricated in this study. In the comparison of the tribological performance between pure epoxy resin and bio-inspired BFRE composites, through simple preliminary tribological tests, the decussated fiber alignment significantly enhances the friction and anti-wear properties, see Figure S1. Simultaneously, pure epoxy resin was inferior to composites with unidirectional bamboo fibers in prior research [14]. However, the optimal fiber alignment for maximizing the tribological performance of BFRE composites remains unclear. Therefore, the bio-inspired BFRE composites in this study primarily comprise bamboo fibers arranged in decussated structures and epoxy resin (mainly constituted by bisphenol-A-diglycidyl-ether and chlorosuccinic anhydride). According to the bamboo fiber orientation, the fibers were categorized as “vertical fibers (VFs)” and “parallel fibers (PFs)”. By controlling the proportion of VFs on the surface of the BFRE composites, this study further investigates the influence of VF proportion of the BFRE composites on its tribological behaviors through systematic morphological analysis, hardness tests, and reciprocating wear tests, which may help provide theoretical guidance for the development of high-performance and green friction materials.

Obvious differences in Vickers hardness between the BFRE composites with different fiber structures are evident in this study. As shown in Figure 6, the existence of decussated structures effectively improved the hardness of the BFRE composites as compared to VFP0% and VFP100%, in which all fibers are uniformly orientated. Clearly, the average hardness of VFP67% was the highest among all kinds of the BFRE composites, even though there are no significant differences between VFP33%, VFP50%, and VFP67%. The observations suggest that the proportion of VFs significantly influences the Vickers hardness of the BFRE composites’ surface. And it can be deduced that the BFRE composites with appropriate decussated structures, such as 33%, 50%, or 67% VF proportion, exhibit stronger resistance to plastic deformation under external loading. Considering that the mechanical properties of fiber-reinforced composites to some extent affect the tribological properties and many friction materials are selected primarily based on this [26,27,28], an appropriate VF proportion of the bio-inspired decussated structures must have a positive impact on the mechanical properties and tribological performances of the BFRE composites.

The different VF proportions of the BFRE composites result in distinct friction behaviors. During the break-in phase, the COF progressively increases, and as the load increases, the duration of the break-in period gradually lengthens (Figure 7a–c). This is likely due to stronger normal loads causing greater material shear, which strips or disperses more fibers, leading to a longer break-in time to achieve a stable state. In Figure 7d, the higher the VF proportion of the surfaces of the BFRE composites is, the lower the average COF becomes. These results are consistent with the literature [29], in which a surface with vertical fibers had a lower COF than one with parallel fibers. And Equation (3), which is applicable to fiber-reinforced polymer materials, from the literature [29] can be used to explain this finding:

(3) $$\frac{1}{\mu }=\frac{V_{f}}{\mu_{f}}+\frac{V_{m}}{\mu_{m}}$$

where *μ* is the overall COF; *V_f_* and *V_m_* are the volume fractions of fiber and matrix, respectively; *μ_f_* and *μ_m_* are the friction coefficients of the fiber and matrix, respectively.

With the VF proportion increased from 0% to 67%, the volume fractions of fibers that participate in friction were reduced, thus decreasing the overall COF. Simultaneously, the correlated friction behaviors can be found in Figure 10. As the VF proportion increased from 25% to 67% (Figure 9b–e), the number of defects, such as the formation and propagation of cracks and the pull-out of fibers, on the worn surfaces of the BFRE composites gradually reduced, indicating a lowering overall COF. Conversely, an excessive proportion of PFs results in most being pulled out and dispersed across the worn surface. This increases the fiber-related volume fraction, amplifying the fiber-associated friction component. With the proliferation of grooves, cracks, and wear debris, an overall increase appears in the system’s friction coefficient, especially under higher loading conditions. Therefore, when the VF proportion reached 75% and 100% under the loads of 10 N and 20 N, substantial resin tearing occurred, exposing the fibers previously covered by resin on the surface (Figure 10f,g), thereby increasing surface roughness and the COF (Figure 7). It indicates that the vertical fibers are sensitive to the external loading, so that under a high loading condition, there exists a mostly appropriate VF proportion for the bio-inspired BFRE composites.

**Figure 11:**
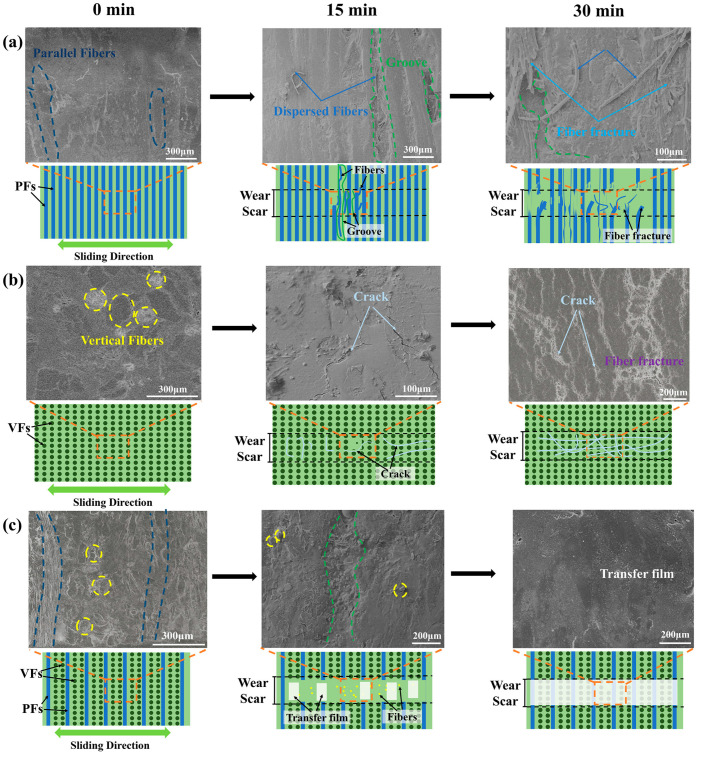
Non-in situ wear process of the BFRE composite material in three different surfaces: (**a**) VFP0%; (**b**) VFP67%; (**c**) VFP100%.

To better understand the wear process, non-in situ wear tests for VFP0%, VFP100%, and VFP67% were conducted in this stage. The SEM morphologies of the worn surfaces and the schematic diagram of the wear mechanisms are shown in Figure 11. As shown in Figure 11a, for the BFRE composites with the fibers all parallel, most parallel fibers can still provide shear resistance support even after 15 min of wear testing. But resin tearing appeared, exposing the fibers to directly suffering the shear forces, which leads to grooves on the surfaces. Height differences on the worn surface due to these grooves were thus formed, increasing surface roughness and leading to the loss of fiber support and exacerbating the wear [14]. As the testing duration lengthened, fibers began to be dispersed, sheared, and pulled out more severely. Those fibers mixed with torn resin to form debris that adheres to the worn surface, further decreasing the shear resistance and increasing roughness, COF, and wear loss [30]. When it comes to the BFRE composites composed entirely of vertical fibers (VFP100%), as shown in Figure 11b, the fibers are arranged perpendicular to the sliding direction, which can reduce the contact area and shear force, thereby reducing wear [31]. But the sliding ceramic ball causes vertical fibers to compress against each other, leading to cracks and fractures on the worn surface. The presence of surface cracks reduces the surface’s ability to resist material removal. And the longer the duration is, the more cracks can be observed on the worn surfaces, causing an increase in the COF and wear rate. Figure 11c depicts the wear process of VFP67%, in which the fibers are decussated, which has been reported to have the ability to enhance the interfacial bonding between fibers and matrix [32], which can further improve the wear resistance. As can be seen, grooves caused by wear occurred on the worn surface after 15 min of wear testing, exposing the parallel fibers, which can be easily removed from the surface, but the addition of vertical fibers reduced the fiber loss as they provide a framework role for the BFRE composites. The absence of cracks, compared to the VFP100% worn surface at the same stage (Figure 11b), indicates that the decussated fiber structure effectively hinders crack initiation and propagation, which is consistent with the function of the fiber structure in bovine tooth enamel [20]. Meanwhile, discontinuous transfer films were observed on the worn surface that covers the fibers along the sliding path, transforming wear behavior from “hard-on-hard” to an intelligent “soft overcoming hard” mode that can reduce the friction coefficient and wear loss [26,31,33,34]. The transfer film became continuous after a longer duration of wear testing, thereby providing an additional anti-wear effect for the BFRE composites. In sum, the bio-inspired fiber decussation has the potential to reduce the wear loss of the BFRE composites compared to the BFRE composites with the fibers all parallel or vertical.

The results of the average specific wear rate shown in Figure 8 also proved that the BFRE composites with bio-inspired structures have better wear resistance. Furthermore, the most appropriate VF proportion of the BFRE composites was found to be 67%. As can be seen, VFP67% has the lowest wear loss among all kinds of the BFRE composites, which can be attributed to the synergetic effect of VFs and PFs on the surfaces of the BFRE composites. As shown for the worn surface profiles in Figure 9, compared to VFP0% (all parallel, Figure 9a) and VFP100% (all vertical, Figure 9g), the wear depths in PF/VF regions decreased significantly once 25% of fibers on the surface were replaced by the fibers with the opposite orientation (Figure 9b,f). And as shown for the worn surface morphologies in Figure 10e, the cracks and grooves disappeared compared to VFP0% (Figure 10a) and VFP100% (Figure 10g). And the wear depths in both the PF and VF regions reached their lowest level when the VF proportion was 67% (Figure 9h), which is consistent with the wear rate results (Figure 8).

By comparing the wear resistance values of all bio-inspired BFRE composites, it can be deduced that an excessive or insufficient VF proportion will decrease the composites’ anti-wear properties. Wear resistance may be conceptualized as a function jointly determined by “in-plane properties” and “interlaminar properties”. In this study, the in-plane properties are mainly contributed by the parallel fibers since they primarily withstand external forces through tension and shear, whilst the interlaminar properties are mainly contributed by the vertical fibers due to the “compression–rebound” effect [35]. Fibers orientated in the same direction as the normal loads can more effectively withstand pressure [34], which can transform the “sliding friction” that causes severe wear into the more advantageous “compression–rebound” process. In the process of reciprocating wear tests, vertical fibers undergo slight elastic deformation once they contact the ceramic ball, but once the ball moves away, the fibers partially rebound. As the overall wear resistance can be seen as the product of the in-plane and the interlayer properties, it will reach a relatively high/low level when the PF/VF ratio is higher/lower than 1:2. This balance is consistent with the decussated structure of bovine enamel, in which the vertical fibers are slightly more abundant than the parallel fibers [19,20]. At this balance, interlayer properties receive disproportionate enhancement without a significant reduction in in-plane properties [35]. This amplified multiplier effect applied to in-plane properties enables the overall wear resistance to reach its maximum value, which helps explain its optimal tribological performance compared to other groups (Figure 7 and Figure 8).

In exploring materials for potential use in the field of friction materials, it is indispensable to consider the stability of the coefficient of friction under diverse and complex real-world friction conditions, which has not been fully addressed in this study. And the preparation process of the BFRE composites in this study makes it difficult to conduct more macroscopic mechanical property tests, such as the tensile test for vertical tension and the flexural test for bending force performance, and the Vickers hardness test can only be used as a plain recognition for the mechanical performance. Despite these limitations, this study reveals that the decussated fiber structure, inspired by bovine enamel, is a highly effective design for enhancing the tribological performance of bamboo-fiber-reinforced composites. And an optimal parallel-fiber/vertical-fiber (PF/VF) ratio of 1:2, at which the bio-inspired BFRE composites exhibit greater anti-wear properties compared to other ratios, was also presented. Within the scope of existing research, these results provide an in-depth analysis of the role of VF proportion in the wear mechanisms of BFRE composites. The findings of this study expand our understanding of the correlation between the bovine-tooth-like decussated structure and its associated tribological mechanisms, thereby offering essential guidance for the biomimetic design of high-performance bamboo-fiber-reinforced composites for applications in friction materials. Our future research will investigate more advanced preparation techniques and the influences of VF proportion on the macroscopic mechanical properties and the stability of the COF of the bio-inspired BFRE composites under diverse friction conditions, including the working conditions of multiple kinds of friction materials.

## 5. Conclusions

This study investigated the influences of the vertical fiber proportions of bio-inspired BFRE composites with decussated fiber structures on their tribological behaviors from the perspectives of surface hardness and wear mechanisms. The main conclusions are summarized as follows:

(1) The bio-inspired fiber decussation can reduce the wear loss of the BFRE composites compared to BFRE composites with the fibers orientated uniformly. Among all the bio-inspired BFRE composites, those with the proportion of 67% vertical fibers (VFP67%) obtain the best wear resistance.
(2) The vertical fibers in the BFRE composites can effectively withstand pressure to enhance the hardness and provide a “compression–rebound” effect, while the parallel fibers provide resistance against shear stress. And the decussated structure effectively inhibits crack initiation and propagation during the wear process and helps facilitate the formation of a transfer film that can reduce the wear loss.
(3) The optimal PF/VF ratio of the decussated fiber structure in the BFRE composites is 1:2; an excessive or insufficient VF proportion will decrease the anti-wear properties.

## Figures and Tables

**Table 1:** Mass percentage of the BFRE composites.

Material	Bisphenol-A-Diglycidyl-Ether	Chlorendic Anhydride	HK-66	Bamboo Fibers
Mass Percentage/wt%	33.8	33.8	2.36	30

**Table 2:** Specimens used in the hardness test and wear test.

Group Name	VF/PF Ratio of Testing Surface	Hardness Test		Wear Test			
		Duration and Load	Number of Samples	Reciprocating Stroke	Frequency and Duration	Loads	Number of Samples
VFP0%	All PFs	20 s and 0.49 N	10	0.4 mm	5 Hz and 30 min	5/10/20 N	12
VFP25%	1/3		10				12
VFP33%	1/2		10				12
VFP50%	1/1		10				12
VFP67%	2/1		10				12
VFP75%	3/1		10				12
VFP100%	All VFs		10				12

## Data Availability

The original contributions presented in this study are included in the article/Supplementary Materials. Further inquiries can be directed to the corresponding author.

## Supplementary Materials

The following supporting information can be downloaded at https://www.mdpi.com/article/10.3390/polym17202765/s1: Figure S1: Comparison of the tribological performance between pure epoxy resin and bio-inspired BFRE composites.
