# Supplementary material for: Research on Bio-Inspired Decussated Bamboo-Fiber-Reinforced Epoxy Composites: The Effect of Vertical Fiber Proportion on Tribological Performances
Source: Polymers (Basel). 2025 Oct 15;17(20):2765. doi: 10.3390/polym17202765 (PMC12566835; doi:10.3390/polym17202765)
Supplement: Supplementary file 1 [file polymers-17-02765-s001.zip › polymers-3892739-supplementary.pdf]

Supplementary material

Research on the bio-inspired decussated bamboo fibers rein-forced epoxy composites: the effect of the vertical fiber proportion in the tribological performances

Heng Xiao, Hao Yi, Zijie Zhou, Ningfeng Wu, Shengwei Liang, Lei Ma*, Wen Zhong

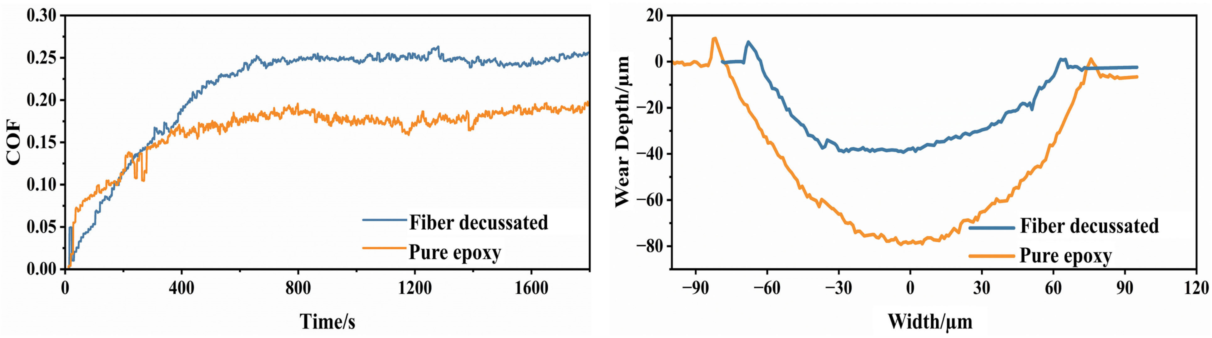

Figure S1. Comparison of the tribological performance between pure epoxy resin and bio-inspired BFRE composites.
